# Characterization of Vimentin-Immunoreactive Astrocytes in the Human Brain

**DOI:** 10.3389/fnana.2020.00031

**Published:** 2020-07-30

**Authors:** Liam Anuj O’Leary, Maria Antonietta Davoli, Claudia Belliveau, Arnaud Tanti, Jie Christopher Ma, William Todd Farmer, Gustavo Turecki, Keith Kazuo Murai, Naguib Mechawar

**Affiliations:** ^1^McGill Group for Suicide Studies, Douglas Mental Health University Institute, Verdun, QC, Canada; ^2^Integrated Program in Neuroscience, McGill University, Montreal, QC, Canada; ^3^Centre for Research in Neuroscience, Department of Neurology and Neurosurgery, Brain Repair and Integrative Neuroscience Program, The Research Institute of the McGill University Health Center, Montreal General Hospital, Montreal, QC, Canada; ^4^Department of Psychiatry, McGill University, Montreal, QC, Canada

**Keywords:** human, mouse, astrocyte, vimentin, GFAP, stereology, morphology

## Abstract

Astrocytes are commonly identified by their expression of the intermediate filament protein glial fibrillary acidic protein (GFAP). GFAP-immunoreactive (GFAP-IR) astrocytes exhibit regional heterogeneity in density and morphology in the mouse brain as well as morphological diversity in the human cortex. However, regional variations in astrocyte distribution and morphology remain to be assessed comprehensively. This was the overarching objective of this postmortem study, which mainly exploited the immunolabeling of vimentin (VIM), an intermediate filament protein expressed by astrocytes and endothelial cells which presents the advantage of more extensively labeling cell structures. We compared the densities of vimentin-immunoreactive (VIM-IR) and GFAP-IR astrocytes in various brain regions (prefrontal and primary visual cortex, caudate nucleus, mediodorsal thalamus) from male individuals having died suddenly in the absence of neurological or psychiatric conditions. The morphometric properties of VIM-IR in these brain regions were also assessed. We found that VIM-IR astrocytes generally express the canonical astrocytic markers Aldh1L1 and GFAP but that VIM-IR astrocytes are less abundant than GFAP-IR astrocytes in all human brain regions, particularly in the thalamus, where VIM-IR cells were nearly absent. About 20% of all VIM-IR astrocytes presented a twin cell morphology, a phenomenon rarely observed for GFAP-IR astrocytes. Furthermore VIM-IR astrocytes in the striatum were often seen to extend numerous parallel processes which seemed to give rise to large VIM-IR fiber bundles projecting over long distances. Moreover, morphometric analyses revealed that VIM-IR astrocytes were more complex than their mouse counterparts in functionally homologous brain regions, as has been previously reported for GFAP-IR astrocytes. Lastly, the density of GFAP-IR astrocytes in gray and white matter were inversely correlated with vascular density, but for VIM-IR astrocytes this was only the case in gray matter, suggesting that gliovascular interactions may especially influence the regional heterogeneity of GFAP-IR astrocytes. Taken together, these findings reveal special features displayed uniquely by human VIM-IR astrocytes and illustrate that astrocytes display important region- and marker-specific differences in the healthy human brain.

## Introduction

Astrocytes constitute approximately 20% of glial cells in the human neocortex, although they vary considerably in abundance throughout this region (Pakkenberg et al., 2003; Pelvig et al., 2008; von Bartheld et al., 2016). Astrocytes have many functions that regulate the central nervous system (CNS), including coupling neuronal activity to blood flow, providing metabolic support and neurotransmitters to neurons, reuptaking neurotransmitters from the synaptic cleft, and controlling the proliferation and repair of neurons (McKeon et al., 1991; Pellerin and Magistretti, 1994; Garcia et al., 2004; Takano et al., 2006; Choi et al., 2012; Covelo and Araque, 2018). Despite their ubiquity and importance, the distributional and morphological features of astrocytes in the human brain have not been extensively characterized, particularly in non-cortical regions. Elegant work by the Nedergaard group has highlighted that human cortical astrocytes display larger cell bodies and more numerous and extensively branched processes than their rodent counterparts (Oberheim et al., 2009). These investigators also described astrocyte subtypes in human cortical gray matter (interlaminar and varicose projection) that are absent in rodents (Oberheim et al., 2009). More recently, Sosunov et al. (2014) described a similar diversity of astrocytes in the human hippocampus. Little information has been published on non-cortical astrocytes, however, and a systematic comparison of astrocyte densities and morphological properties across human brain regions has yet to be published. Such work has been done for mouse astrocytes, which exhibit substantial regional heterogeneity in their density and morphology (Emsley and Macklis, 2006; Eilam et al., 2016), and their functional interactions with neurons (Morel et al., 2017). Moreover, regional differences in the distribution and function of astrocytes in the mouse brain are developmentally patterned and actively maintained by their neighboring neurons (Magavi et al., 2012; Gao et al., 2014; Farmer et al., 2016). The regional patterning of astrocytes likely coordinates their functional roles in neuronal circuits, given that mouse astrocytes can selectively contact specific subtypes of neurons and mediate region-specific functions (Suzuki et al., 2011; Perea et al., 2014; Martín et al., 2015; García-Cáceres et al., 2016).

The cytoskeleton of astrocytes is characteristically defined by the relatively high expression of three components: actin, glial fibrillary acidic protein (GFAP), and vimentin (VIM) (Chiu et al., 1981). Both VIM and GFAP share functional properties, as they are type III intermediate filaments predominantly found in astrocytes and upregulated in cells undergoing reactive astrogliosis (Pixley and de Vellis, 1984). Moreover, mouse astrocytes that lack both VIM and GFAP have impaired glial scar formation and decelerated immune response at the level of vesicle trafficking, indicating that VIM and GFAP regulate how astrocytes responses to inflammation and can functionally compensate for each other (Wilhelmsson et al., 2004; Potokar et al., 2010; Vardjan et al., 2012). However, VIM appears to have more regulatory roles for astrocyte structure, as the translation of two other intermediate filaments common to astrocytes — nestin and synemin — require VIM, but not GFAP (Pekny et al., 1999; Jing et al., 2007). Moreover, there is a reciprocal relationship between the relative expression of VIM and GFAP with age, such that prior to cortical myelination, developing astrocytes have high VIM and low GFAP expression, whereas in adulthood, mature astrocytes have low VIM and high GFAP expression (Dahl, 1981). This may be why most morphological descriptions of mature astrocytes, in both model species and humans, have been largely based on the analysis of cells immunoreactive (-IR) for GFAP, but not VIM. As in the mouse brain, there may be substantial regional heterogeneity of GFAP-IR astrocytes in the human brain, given that there are significant inter-regional differences in GFAP mRNA and protein expression which appear qualitatively related to the distribution of human GFAP-IR astrocytes (Torres-Platas et al., 2016). In contrast to GFAP-IR astrocytes, little is known of the general properties or regional heterogeneity of astrocytes in adult CNS tissues, even though they were observed in postmortem human brain tissue nearly three decades ago (Yamada et al., 1992).

Based on our preliminary observations in adult human cerebral cortex that VIM clearly labels astrocytic cell bodies and their processes (unpublished data), and that VIM-IR and GFAP-IR astrocytes are both abundant, we pursued a more systematic analysis of VIM-IR astrocytes in different regions of the human brain. Here, we report the first quantitative regional data on human VIM-IR astrocytes. More specifically, we determined the densities and morphometric properties of VIM-IR astrocytes in four regions of the adult human brain: prefrontal cortex, primary visual cortex, caudate nucleus and dorsomedial thalamus. We selected the same cortical and subcortical regions we previously used in regional comparisons of astrocytic markers (Torres-Platas et al., 2016; Nagy et al., 2017). The visual cortex was of particular interest as it presents interlaminar astrocytes, a primate-specific subtype of astrocyte (Oberheim et al., 2009). In addition to co-immunolabeling experiments to determine the overlap of VIM-IR astrocytes with two other canonical astrocyte markers — GFAP and Aldh1L1 — we generated similar data for mouse VIM-IR astrocytes which allowed for cross-species comparisons. Finally, we assessed whether the regional heterogeneity in vascular density was associated with that of astrocyte density and morphometry. This study is the first to characterize VIM-IR astrocytes in adult human and mouse brains and to quantify the extent of regional heterogeneity for astrocytes across regions, markers and species.

## Materials and Methods

### Brain Samples

This study was approved by the Douglas Hospital Research Ethics Board. Brains were donated to the Douglas-Bell Canada Brain Bank by familial consent through the Quebec Coroner’s Office, which ascertained the cause of death. Brain samples from 10 Caucasian male individuals having died suddenly without any known inflammatory, psychiatric or neurological disorder were analyzed (Table 1). Four brain regions were dissected from freshly received specimens of mediodorsal thalamus, dorsal caudate nucleus (precommissural level), and two cortical areas: prefrontal cortex [Brodmann area (BA) 8/9] and primary visual cortex (BA17).

**TABLE 1:** Subject information.

	Subject information
Age	57.0 ± 6.9
Sex	10M
Tissue pH	6.3 ± 0.1
Postmortem interval (hours)	54.8 ± 9.2
Cause of death	6 natural, 4 accidental

Brain samples were also used from five adult male C57BL/6 mice (Charles-River Canada) which served as controls in a previous study (sub-chronic subcutaneous saline administration; Mahar et al., 2011) approved by McGill University’s Animal Care Committee (MACC approval ID: 5473). Four mouse brain regions were studied: the mediodorsal thalamus, the caudate-putamen (CPu, precomissural level), the frontal association cortex (FrA) and primary visual cortex (V1). Human BA8/9 and BA17 were seen as homologous to FrA and V1, respectively.

Furthermore, we examined three brains from adult *Aldh1L1*-Cre/ERT2; *Rosa26-TdTomato* transgenic mice (Montreal General Hospital Animal Use Protocol ID: 6005) generated by crossing Aldh1L1-Cre/ERT2 mice (JAX stock no. 031008) with Ai9 Rosa26-TdTomato reporter line mice (JAX stock no. 007909) (Madisen et al., 2010; Srinivasan et al., 2016). This Cre reporter strain enables highly specific targeting of TdTomato fluorescence in astrocytes in adult mouse CNS with a stronger signal and less background than antibody staining, and reveals over 90% of astrocytes immunolabelled with the astrocyte marker S100β (Winchenbach et al., 2016). All mice were group-housed on a 12:12 light:dark cycle with *ad libitum* access to food and water and sacrificed at 2 months of age (see below) following the policies and guidelines of the Canadian Council on Animal Care.

### Tissue Processing

For 8 of the 10 human subjects, fresh 1 cm^3^ blocks of cerebral tissue were fixed overnight in 10% neutral buffered formalin at 4°C, suspended in 30% sucrose solution until equilibrium was reached, flash frozen in -35°C isopentane, and cut on a sliding microtome into 50 μm-thick serial sections that were stored at -20°C in a cryoprotectant solution until they were processed for immunohistochemistry (IHC). For the remaining 2 subjects, samples were processed in the same way except that they were cut on a cryostat into 10 μm-thick serial sections until they were processed for double-labeling immunofluorescence (IF) and fluorescent *in situ* hybridization (FISH).

As described previously (Mahar et al., 2011), mice were anesthetized using a mixture of ketamine (50 mg/kg, Vetrepharm, Canada), xylazine (5 mg/kg, Novopharm, Canada) and acepromazine (0.5 mg/kg, Ayerst, Canada), then perfused intracardially with ice-cold phosphate-buffered saline (PBS) followed by 4% formaldehyde in 0.1 M phosphate buffer. Brains were rapidly removed, fixed and suspended in sucrose as above before being cut coronally into 40 μm-thick serial sections on a cryostat and stored in cryoprotectant at -20°C until they were processed for IHC and IF.

### Immunolabeling

All IHC and IF procedures were conducted at room temperature with three 5 min washes in phosphate-buffered saline (PBS) after each step except for after the pre-incubation blocking step. For IHC, sections were incubated for 20 min in 3% H_2_O_2_ to quench endogenous peroxidase activity, blocked for 1 h in PBS containing 0.2% Triton-X (PBS-Tx-100) and 10% normal serum from the secondary antibody host species, and incubated overnight in PBS-Tx-100 containing 2% normal serum and primary antibody (Table 2). On the following day, sections were incubated in biotinylated secondary antibodies (Jackson; 1:500) for 1 h, and then in avidin-biotin-peroxidase complex (Vector) for 30 min. Immunostaining was revealed by a 3 min incubation with a 3-3′-diaminobenzidine (DAB) peroxidase substrate kit (Vector Laboratories, SK-4100). Sections were mounted onto glass slides, dried overnight, dehydrated in a graded ethanol series, cleared in xylene, and coverslipped with Permount medium (Fisher Scientific). For double-labeling IF experiments, sections were incubated with both rabbit anti-VIM and chicken anti-GFAP primary antibodies to be bound respectively to secondary Alexa Fluor 488- and Cy5-conjugated antibodies (Thermo Fisher). Sections were mounted onto glass slides and coverslipped in DAPI mounting medium (Vector).

**TABLE 2:** Antibodies used.

Primary antibody	Species	Clone	Dilution	Source
Vimentin	Rabbit	ab92547	1:500 (IHC-DAB) 1:250 (IF)	Abcam
GFAP	Chicken	ab4674	1:1000 (IHC-DAB) 1:1000 (IF)	Abcam
CD31	Mouse	JC70	1:250	Santa Cruz

For the IF experiment using transgenic mice, we assessed VIM-immunofluorescent cells in the mouse CPu, as in our sections it was the largest mouse brain region that was homologous to one of the human brain regions in this study. To assess whether VIM was commonly found in mouse GFAP-IR astrocytes and the extent to which it was expressed in all astrocytes in a given brain region, we made use of an available mouse genetic line for the astrocyte marker Aldh1L1. Of all known astrocyte markers to date, Aldh1L1 is the best for representing the majority of astrocytes, as it is expressed in the largest number of astrocytes. Transgenic animals were needed to assess the proportion of all astrocytes (as best revealed by Aldh1L1) that express VIM, because Aldh1L1 immunostaining is particularly challenging in mouse brain tissue (unpublished observations). Transgenic fluorescent reporter proteins also reveal the entire cell morphology, enabling us to qualitatively compare the extent of morphology revealed by VIM immunolabelling. Moreover, the increased strength and specificity of fluorescent reporter proteins over immunolabelling approaches facilitated a more precise localization between astrocytes expressing both VIM and Aldh1L1 proteins.

### Fluorescent *in situ* Hybridization (FISH)

To clearly confirm that human VIM-IR cells are astrocytes, we labeled Aldh1L1 expression, as it is the most widely and homogeneously expressed astrocytic marker (Cahoy et al., 2008). An RNAScope Multiplex Fluorescent v2 kit (Advanced Cell Diagnostics, Newark, CA, United States) with ALDH1L1 (catalog no. 438881) and GFAP (catalog no. 311801-C2) mRNA probes was used in combination with IF to label VIM (using the same protocol for VIM IF as in double-labeling experiments). Fresh-frozen sections (10 μm-thick) of prefrontal cortex were mounted onto charged glass slides, and stored at -80°C until the FISH assay was performed according to the manufacturer’s instructions. Following the FISH protocol, a standard IF protocol was performed on slides, which involved an overnight incubation in primary antibody solution and an hour-long incubation in secondary antibody solution on the following day prior to coverslipping with mounting medium containing DAPI.

### Double-Labeling Analysis

To assess the cellular co-localization of VIM protein with canonical astrocyte markers, images were acquired at 40X using a FV1200 laser scanning confocal microscope equipped with a motorized stage (Olympus, Japan). Sections from the caudate nucleus and prefrontal cortex were used, and as they were thin (10 μm thick), single acquisitions were performed instead of *z*-stacks to increase the efficiency of sampling. Cells were identified as GFAP+, VIM+ or GFAP+/VIM+. Data was expressed as the mean proportion of GFAP+ cells that were also VIM+ (GFAP+/VIM+), and the mean proportion of VIM+ cells that were also GFAP+ (VIM+/GFAP+). The same method was used for comparing VIM and Aldh1L1 co-localization. Per subject and region, GFAP protein co-localization was assessed in at least 85 VIM-IR cells, GFAP RNA co-localization was assessed in at least 70 VIM-IR cells, and Aldh1L1 RNA co-localization was assessed in at least 100 VIM-IR cells. For mouse co-localization analysis, a similar approach was used on all VIM-IR cells imaged within the CPu of one hemisphere in all three transgenic mice.

### Stereological Cell Counting

VIM-IR and GFAP-IR astrocytes were counted using DAB immunolabelling visualized with brightfield microscopy on a workstation connected to a BX51 microscope equipped with a motorized stage and CX9000 camera (Olympus, Japan). An unbiased stereological approach was performed using StereoInvestigator software (MBF Bioscience, United States) and average regional astrocyte densities were calculated by dividing the total regional population estimate (obtained with the Optical Fractionator probe) by the total regional volume (obtained with the Cavalieri probe). For each subject and region counted, we selected four equally spaced sections from a series consisting of every twelfth section of the region of interest. We chose four sections as, across all subjects and regions, this was the largest stereological series available, and the largest number of sections that could fit on a single slide (preventing potential differences in mounted thickness between slides for the same subject and region). A contour was drawn around the perimeter of each section avoiding sectioning and staining artifacts. The Optical Fractionator Probe facilitates unbiased counting by randomly assigning counting frames at either a consistent number of sites or that cover a consistent area of the section contour. For this study, we chose to sample a consistent percentage area, to ensure the same proportion was sampled for each slide despite considerable differences in section area between subjects and regions. Stereological counting rules were applied to the cell body of strongly stained cells with astrocytic morphology. A pilot study was performed on to identify the largest dissector height (18 μm with 1 μm guard zones) and smallest contour sampling size (5%) with which the probe could then provide total regional population estimates with an accurate Gundersen coefficient of error (CE, *m* = 1) < 0.10. This pilot study was repeated for all objective magnifications at which astrocytes could be reliably distinguished with both markers — X40, X60, and X100. As all magnifications produced similar results, X40 magnification was chosen to increase the speed of counting. After all regional cell number estimates were acquired, the Cavalieri Estimator probe was used on the same contours used for cell counting (100 μm grid spacing). These parameters for regional density estimates were sufficient to account for the heterogeneous distribution of astrocytes within each region, as there was very little difference between estimates achieved using the same parameters within the same contours in the pilot study.

### Morphometric Features

With the same slides and workstation used for stereological analysis, the fine morphometry of representative VIM-IR astrocytes was manually traced live using a computer-based tracing system with a 100X oil immersion objective and Neurolucida software (MBF Bioscience, United States). We employed manual 3D tracing as we have previously shown this method to be effective at revealing intricate differences in the fine morphology of human astrocytes (Torres-Platas et al., 2011). Briefly, the entire slide was scanned at 10X objective magnification and the location of representative cells for tracing was digitally recorded with respect to a reference point. Representative cells were identified and had to be: (1) unobstructed by neighboring cells; (2) similarly sized to neighboring cells; (3) equally stained across cellular compartments; (4) contained within the thickness of the section; (5) in contact with VIM-IR blood vessels via clear endfeet contacts. After switching to the 100X oil immersion objective, the locations were revisited in chronological order, and the first four cells that continued to fulfill the criteria for representative cells at 100X magnification were traced. A biased sampling method for selecting representative cells was needed due to the low number of cells traced, and to avoid inconsistencies in the quality of VIM-IR immunostaining evident only at high magnification that prevent precise reconstructions. The cell body was reconstructed by drawing a single ring around the perimeter while maintaining focus in the *z*-axis. Processes were then selected for tracing in a clockwise manner; the length and width of each process was traced outward from the cell body toward its terminals in *XYZ* coordinates, including each branch point (node). For human astrocyte morphometry, cortical gray and white matter were analyzed separately, resulting in four cells being reconstructed in six areas of five subjects (Table 1), for a total of 120 VIM-IR cells. For mouse astrocyte morphometry, four cells were analyzed in three areas of five animals, for a total of 60 mouse VIM-IR cells. A branched Structure Analysis (BSA) was performed on all reconstructed cells using Neurolucida to compare essential structural features of astrocyte morphology. These include the mean number of primary processes (process number), the mean number of process branch points (node number) and the mean number of process end points (terminal number), and the size and area of processes and cell bodies.

### Vascular Density

As the processes of VIM-IR astrocytes clearly contacted VIM-IR blood vessels that qualitatively varied in density across regions, the vascular density was quantified to assess whether it correlated with the density or morphometry of VIM-IR astrocytes. For reliable estimates of vascular density, we immunolabelled a receptor called cluster of differentiation 31 (CD31), also known as platelet endothelial cell adhesion molecule (PECAM-1), that is abundantly expressed in vascular endothelium (Ogunshola et al., 2000). Sections were prepared as for stereology and morphology and to estimate the vascularization of each region, CD31- and VIM-IR blood vessels were imaged in a random systematic manner using the SRS Image Series workflow in StereoInvestigator software (MBF Bioscience, United States). All images were taken at low (10X objective) magnification, and 5% of the area of each section immunolabeled for stereology was imaged. The area occupied by blood vessels was determined by manually drawing the contours of blood vessels using the ROI manager of the ImageJ software in five images per region per subject. In each case, these were the first five images found to contain no observable artifacts or background staining. A similar method was used for CD31-IR blood vessels in the mouse brain, except fewer than 5 images were required to cover 5% of the area of the sections.

### Statistical Analysis

All statistical analyses were performed using Prism v. 6.04 (GraphPad Software, San Diego, CA, United States). Data were assessed for a normal distribution using the D’Agostino and Pearson omnibus normality test. For non-parametric data which compared two groups or regions, two-tailed Mann–Whitney *U* tests were performed. For parametric data which compared more than two groups or regions, group differences were detected using a Matched One-Way ANOVA with multiple comparisons corrected for by the Bonferroni *post hoc* test. Non-parametric data sets obtained using the same samples had within-group differences detected using a standard Friedman Test with multiple comparisons corrected for by Dunn’s *post hoc* test. All measurements are expressed as mean ±SEM, and adjusted *P*-values are reported with a significance threshold of 0.05.

## Results

### Vimentin Immunoreactivity in the Human Brain: General Observations

**FIGURE 1:**
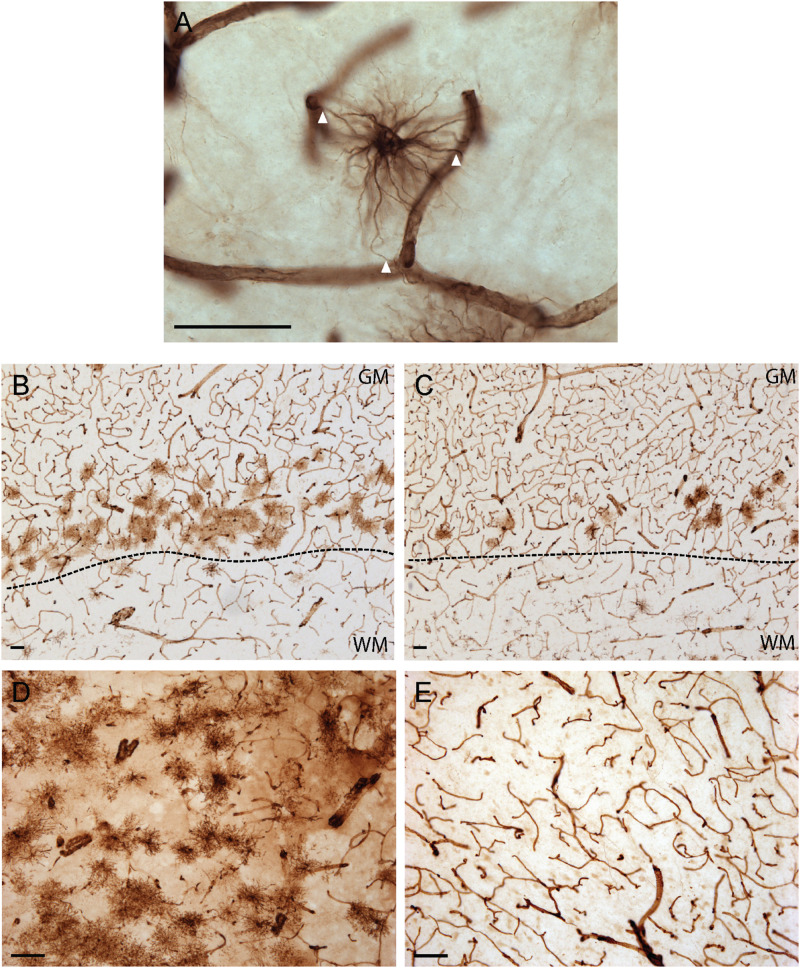
Vimentin immunohistochemistry strongly labels blood vessels and astrocytes in healthy adult human brain. **(A)** Example of a VIM-IR astrocyte in the caudate nucleus juxtaposed to and contacting (arrowheads) VIM-IR blood vessels. **(B)** VIM-IR astrocytes in the prefrontal cortex gray matter outnumbered those in white matter and are clustered along the gray/white matter boundary (dashed line). **(C)** Although distributed in a similar pattern along the gray/white matter boundary (dashed line), fewer VIM-IR astrocytes were observed in the primary visual cortex compared to the prefrontal cortex. **(D)** VIM-IR astrocytes were most abundant in the caudate nucleus. **(E)** VIM-IR astrocytes were almost completely absent from the mediodorsal thalamus. Scale bars = 50 μm.

As illustrated in Figure 1, VIM immunoreactivity was observed to strongly label blood vessels in all human brain regions examined; a pattern consistent with the reported expression of VIM as the principal intermediate filament of endothelial cells (Gabbiani et al., 1981). In addition to vascular immunolabelling, VIM-IR cells with all the morphological attributes of astrocytes — including making contact with a nearby VIM-IR blood vessel (Figure 1A) — were also reliably observed in all regions, with variable densities. For both cortical areas examined, VIM-IR astrocytes were consistently more abundant in gray matter than in white matter. Most protoplasmic astrocytes were localized in deeper gray matter layers and were noticeably aligned above the gray/white matter boundary. We observed more protoplasmic astrocytes in prefrontal (Figure 1B) than in visual cortex (Figure 1C), mostly due to an increased thickness and continuity of this lower-layer band. In the caudate nucleus, VIM-IR astrocytes were densely distributed mainly around large VIM-IR blood vessels. While these astrocytes had a protoplasmic-like morphology, they often had many processes that closely overlapped those of neighboring astrocytes in the *z*-axis, and so were not clearly organized in domains as in the cortex for GFAP-IR astrocytes (Figure 1D). Certain sections of mediodorsal thalamus were completely devoid of VIM-IR astrocytes despite the presence of blood vessels strongly immunostained for VIM (Figure 1E). When present, thalamic VIM-IR astrocytes had a fibrous-like morphology but appeared notably smaller than cortical VIM-IR protoplasmic and fibrous astrocytes. In addition to strongly labeling astrocytes and endothelial cells, VIM-IHC faintly labeled small ramified microglia in cortical white matter, as has been previously reported in postmortem human brain tissue (Yamada et al., 1992). Such VIM-IR microglia were never observed in cortical gray matter nor in subcortical regions, and were not taken into consideration in our cell density and morphometric analyses.

### Vimentin Is Mostly Co-expressed With Other Astrocytic Markers

**FIGURE 2:**
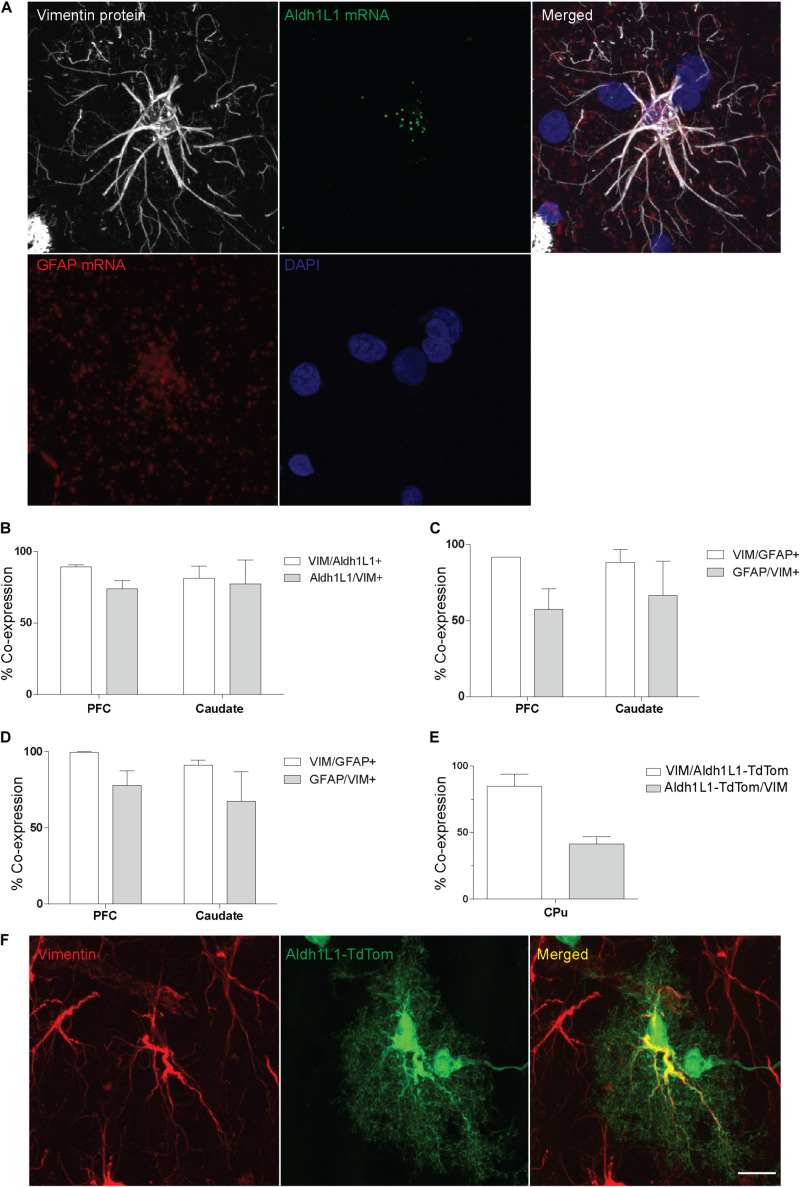
VIM-IR cells express other astrocytic markers in adult human and mouse brain. **(A)** Representative example in the human prefrontal cortex of a VIM-IR astrocyte revealed using immunofluorescence (white), containing Aldh1L1 mRNA (green) and GFAP mRNA (red), revealed using fluorescent *in situ* hybridization (FISH), within the nucleus (DAPI-stained, blue). **(B)** By combining FISH with immunofluorescence, Aldh1L1 mRNA was observed in the majority of DAPI-stained nuclei of VIM-IR astrocytes in the PFC and the caudate nucleus (*n* = 2). **(C)** By combining FISH with immunofluorescence, GFAP mRNA was observed in the majority of DAPI-stained nuclei of VIM-IR astrocytes in the PFC and the caudate nucleus (*n* = 2). **(D)** Using coimmunofluorescence, the majority of astrocytes in the PFC and the caudate nucleus coexpressed VIM protein and GFAP protein (*n* = 2). **(E)** In transgenic adult mice, the fluorescence of cre reporter proteins in astrocytes expressing Aldh1L1 was observed in many VIM-IR astrocytes in the caudate-putamen (CPu) (*n* = 3). **(F)** VIM protein expression (red) was located within thick processes of transgenically Aldh1L1-labeled (green) astrocytes in the mouse CPu. Scale bars = 25 μm.

To further confirm the phenotype of VIM-IR cells as astrocytes, we used FISH and IF in the prefrontal cortex and caudate nucleus to localize the cellular expression of VIM with two commonly used astrocyte markers, Aldh1L1 and GFAP (Figure 2). By combining FISH for both Aldh1L1 and GFAP mRNA probes and immunofluorescence for VIM protein, Aldh1L1 and GFAP mRNA expression was observed within the DAPI-stained nucleus of VIM-IR cells in the prefrontal cortex (Figure 2A). In both regions, more than 80% of VIM-IR astrocytes were positive for Aldh1L1 mRNA, and more than 70% of cells expressing Aldh1L1 mRNA were immunofluorescent for VIM (Figure 2B). Similar findings were made for GFAP mRNA, as 90% of VIM-IR astrocytes co-expressed GFAP mRNA, and more than 60% of astrocytes expressing GFAP mRNA also displayed VIM immunoreactivity (Figure 2C). Co-immunofluorescence was then used to further examine the extent to which VIM protein and GFAP protein are localized to the same cellular population, and in both regions more than 90% of VIM-IR astrocytes were also GFAP-IR, and more than 70% of GFAP-IR astrocytes were VIM-IR as well (Figure 2D). In the mouse CPu, we found more than 80% of VIM-immunofluorescent astrocytes were labeled by Cre-driven reporter protein expression for Aldh1L1, whereas approximately 40% of these transgenically labeled cells were also VIM-immunofluorescent (Figure 2E). In these Aldh1L1-TdTom astrocytes, VIM immunofluorescence labeled thick processes but not the fine perisynaptic astrocytic processes extended by these cells (Figure 2F). Collectively, these observations confirm that most, but not all, VIM-IR astrocytes express the canonical astrocytic markers GFAP and Aldh1L1. Vimentin immunoreactivity labels previously described morphological subtypes of astrocytes.

**FIGURE 3:**
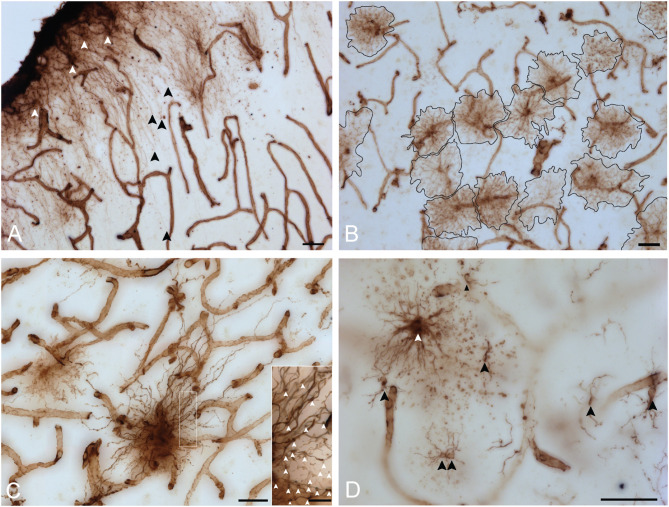
VIM immunohistochemistry labels astrocytic subtypes previously described for GFAP-IR astrocytes in human neocortex. **(A)** Fine unbranched fibers (black arrowheads) emerging from darkly labeled VIM-IR interlaminar astrocytes (white arrowheads) and projecting to layer III. **(B)** VIM-IR astrocytes in the prefrontal cortex gray matter have a non-overlapping domain organization. **(C)** An example of a VIM-IR astrocyte in deep cortical gray matter (prefrontal cortex) with the attributes of a varicose projection astrocyte. The inset shows a magnified view of regularly spaced varicosities (arrows) on VIM-IR varicose projection astrocyte processes. **(D)** In cortical white matter and thalamus, VIM-IR astrocytes (white arrowhead) extended rather straight and mostly unbranched processes, as is typical for GFAP-IR fibrous astrocytes. VIM-IR microglia (black arrowheads) were also observed in cortical white matter. In general, these cells were weekly stained and easily distinguishable from VIM-IR astrocytes. Scale bars = 50 μm.

VIM immunoreactivity revealed all four known morphological subtypes of human cortical astrocytes previously described with GFAP IHC (Oberheim et al., 2009) (Figure 3). In layer I, astrocytes with a unipolar morphology extending long, direct and unbranching processes into layer III were observed, displaying the characteristic features of interlaminar astrocytes (Figure 3A). Although VIM-IR interlaminar astrocytes were clearly visible in primary visual cortex, they were not clearly identifiable in prefrontal cortex — all other morphological subtypes were found in both prefrontal and visual cortex. In the remaining layers (II–VI) of neocortical gray matter, VIM-IR astrocytes displayed the stellate morphology conferred by extensively branched bushy processes that is characteristic of protoplasmic astrocytes (Figure 3B). VIM-IR protoplasmic astrocytes were sparsely distributed and, in areas of high local density, occupied non-overlapping domains. In deep cortical gray matter (layers V-VI) VIM-IR varicose projection astrocytes extending exceptionally long processes bearing regularly spaced varicosities were observed (Figure 3C). These processes more often radiated toward upper layers than toward white matter. Few varicose projection astrocytes were seen, however, particularly in the visual cortex. In cortical white matter, we observed astrocytes with straight, finely tortuous and rarely branching processes, as is characteristic of fibrous astrocytes (Figure 3D).

### Vimentin Immunoreactivity: Regional Astrocytic Features

**FIGURE 4:**
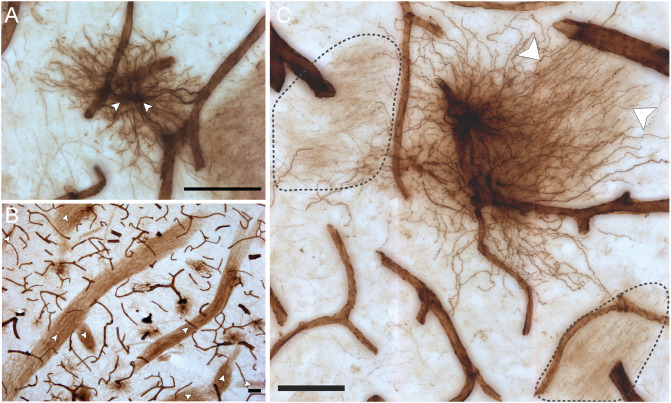
VIM-IR astrocytes display diverse morphological features. **(A)** Two adjacent VIM-IR astrocyte cell somas (arrowheads) displaying a twin cell morphology were commonly observed in all human brain regions examined. **(B)** In the caudate nucleus, we observed long bundles of parallel VIM-IR fibers (arrowheads) of no immediately discernable cell origin or target, but which often received contacts from neighboring VIM-IR astrocytes. **(C)** Some VIM-IR astrocytes extended parallel projections (arrowheads) which may be the origin of the fiber bundles illustrated in **(C)** and which appear (black outline) from outside of the *z*-plane of the section, suggesting that VIM-IR astrocytes contact distal targets. Scale bars = 50 μm.

Some unique regional features were observed for human VIM-IR astrocytes that have not previously been reported following labeling with other astrocyte markers (Figure 4). A high proportion (approximately 20%) of neocortical protoplasmic or striatal protoplasmic-like VIM-IR astrocytes exhibited a twin cell morphology, consisting of two abutting cell bodies apparently formed from astrocyte cell division (Figure 4A); a phenomenon rarely observed for GFAP-IR astrocytes (not shown). Furthermore, long varicose bundles of VIM-IR processes with no identifiable cellular origin were also observed in the caudate nucleus (Figure 4B). These fasciculi appeared to form networks as they seemed connected to each other and received projections from nearby astrocytes (Figures 4B,C). They appeared similar in density to the processes of some VIM-IR astrocytes, which can project many unbranching, parallel processes toward VIM-IR blood vessels (Figure 4C).

### Regional Densities of Vimentin- and GFAP-Immunoreactive Astrocytes

**FIGURE 5:**
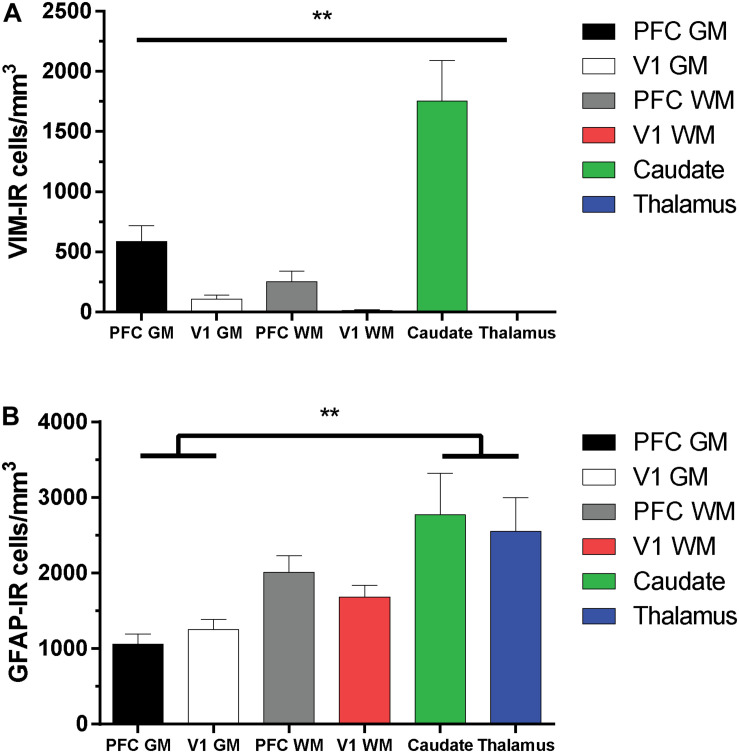
Regional stereological estimates of VIM-IR and GFAP-IR astrocyte density in the adult human brain. **(A)** There were significantly more VIM-IR astrocytes in the caudate nucleus than in all other brain regions examined, including the thalamus where VIM-IR astrocytes were mostly absent. **(B)** All human brain regions included in this study were more densely populated by GFAP-IR astrocytes than by VIM-IR astrocytes. There were also significantly more GFAP-IR astrocytes in either subcortical region than in neocortical areas. **p ≤ 0.01 (*n* = 8; Matched One-Way ANOVA).

As the distribution and features of VIM-IR astrocytes varied across regions, a stereological approach was used to quantify regional differences in astrocyte densities (Figure 5). Strongly significant differences in the regional densities of VIM-IR astrocytes were found (Figure 5A). In cortical gray matter, about five times more VIM-IR astrocytes were observed in the prefrontal cortex than in the primary visual cortex (586 ± 132 cells/mm^3^ vs. 108 ± 32 cells/mm^3^). These differences were even larger in cortical white matter, where fifteen times more VIM-IR astrocytes were observed in the prefrontal cortex than in the primary visual cortex (252 ± 85 cells/mm^3^ vs. 14 ± 4 cells/mm^3^). The caudate nucleus was significantly more densely populated with VIM-IR astrocytes than all other regions studied (1753 ± 339 cells/mm^3^), with nearly three times more VIM-IR astrocytes than in prefrontal cortex gray matter. Caudate astrocytes were often localized in the vicinity of larger blood vessels. VIM-IR astrocytes were rarely observed in the mediodorsal thalamus (1 ± 1 cell/mm^3^), with many immunostained sections containing no VIM-IR astrocytes at all.

For comparison, the density of GFAP-IR astrocytes was assessed in the same subjects and found to be more similar across regions than for VIM-IR astrocytes (Figure 5B). In cortical gray matter, more GFAP-IR astrocytes were found in the visual cortex than in the prefrontal cortex (1256 ± 129 cells/mm^3^ vs. 1059 ± 133 cells/mm^3^). The opposite pattern was found in cortical white matter, where more GFAP-IR astrocytes were found in the prefrontal cortex than in the visual cortex (2011 ± 222 cells/mm^3^ vs. 1686 ± 153 cells/mm^3^). The density of GFAP-IR astrocytes in either the caudate nucleus (2771 ± 551 cells/mm^3^) or the thalamus (2555 ± 448 cells/mm^3^) was significantly greater in the gray matter, but not the white matter, of both cortical regions. As there were more GFAP-IR than VIM-IR astrocytes in all regions, this suggests that VIM reveals a relatively small subset of astrocytes.

### Morphometric Properties of Vimentin-Immunoreactive Astrocytes

**FIGURE 6:**
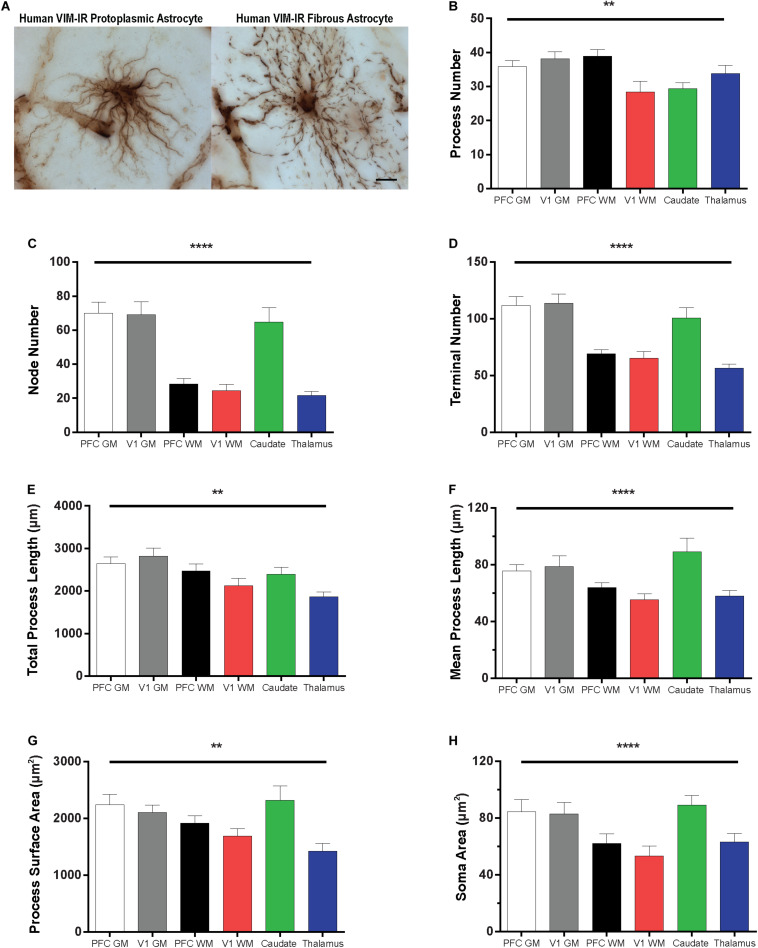
Regional heterogeneity in human VIM-IR astrocyte morphometry. **(A)** Representative VIM-IR astrocytes from prefrontal cortex gray matter (left) and white matter (right). **(B–H)** Branched Structure Analysis (BSA) of VIM-IR astrocytes revealed morphometric differences across markers and regions. BSA measurements for VIM-IR astrocytes were generally similar for cortical gray matter and caudate nucleus, and for cortical white matter and mediodorsal thalamus. Scale bars = 50 μm. **p ≤ 0.01, ****p ≤ 0.0001 (*n* = 5; Friedman Test).

The morphology of VIM-IR astrocytes varied significantly across regions for all branched structured analysis (BSA) features (Figure 6). The process number of VIM-IR astrocytes in visual cortex white matter (28 ± 3 processes) was significantly lower than that in prefrontal cortex white matter (39 ± 2 processes) and visual cortex gray matter (38 ± 2 processes), but no significant differences were found between neocortical gray matter regions or between subcortical regions (Figure 6B). The strongest regional difference for VIM-IR astrocytes was in the number of nodes, which constitute branch points in astrocyte processes (Figure 6C). There was twice as many nodes for VIM-IR astrocytes in the gray matter than in the white matter compartment of prefrontal cortex (70 ± 7 vs. 28 ± 3 nodes) and visual cortex (69 ± 7 vs. 24 ± 4 nodes), and thrice as many nodes for VIM-IR astrocytes in the caudate nucleus than in the mediodorsal thalamus (65 ± 9 vs. 22 ± 3 nodes). No significant differences in VIM-IR node number were found between neocortical gray or white matter compartments. The VIM-IR terminal number had the same pattern of significant differences as for VIM-IR node number (Figure 6D), except that the terminal number in the caudate nucleus (101 ± 9 terminals) did not significantly differ from those found in prefrontal cortex or visual cortex white matter (69 ± 4 and 66 ± 6 terminals, respectively). The total process length of VIM-IR astrocytes was significantly greater only in visual cortex gray matter relative to the mediodorsal thalamus (2820.0 ± 189.2 vs. 1860.0 ± 122.6 μm), confirming qualitative observations of smaller VIM-IR astrocytes in the mediodorsal thalamus (Figure 6E). As for VIM-IR process number, VIM-IR mean process length significantly differed only between visual cortex white matter (55.4 ± 4.1 μm) and the two highest regional values (Figure 6F), in this instance being those for the caudate nucleus and visual cortex gray matter (89.3 ± 9.6 vs. 78.8 ± 7.6 μm, respectively). A similar finding was made for the total process surface area of VIM-IR astrocytes (Figure 6G), which was significantly larger in the caudate nucleus (2321.0 ± 251.9 μm^2^) and prefrontal cortex gray matter (2238.0 ± 183.8 μm^2^) than in the mediodorsal thalamus (1423.0 ± 137.1 μm^2^). The soma area of VIM-IR astrocytes was typically smaller in cortical white matter (Figure 6H), as there was a significant difference in VIM-IR soma area between visual cortex grey and white matter (82.85 ± 8.2 vs. 53.2 ± 7.0 μm^2^), and between the caudate nucleus (89.1 ± 6.9 μm^2^) and either prefrontal or visual white matter (62.0 ± 6.8 μm^2^ and 53.2 ± 7.0 μm^2^, respectively). These observations generally show that cortical VIM-IR astrocytes have a more complex morphology in gray matter than in white matter.

### Comparison of Human and Mouse Vimentin-Immunoreactive Astrocytes

**FIGURE 7:**
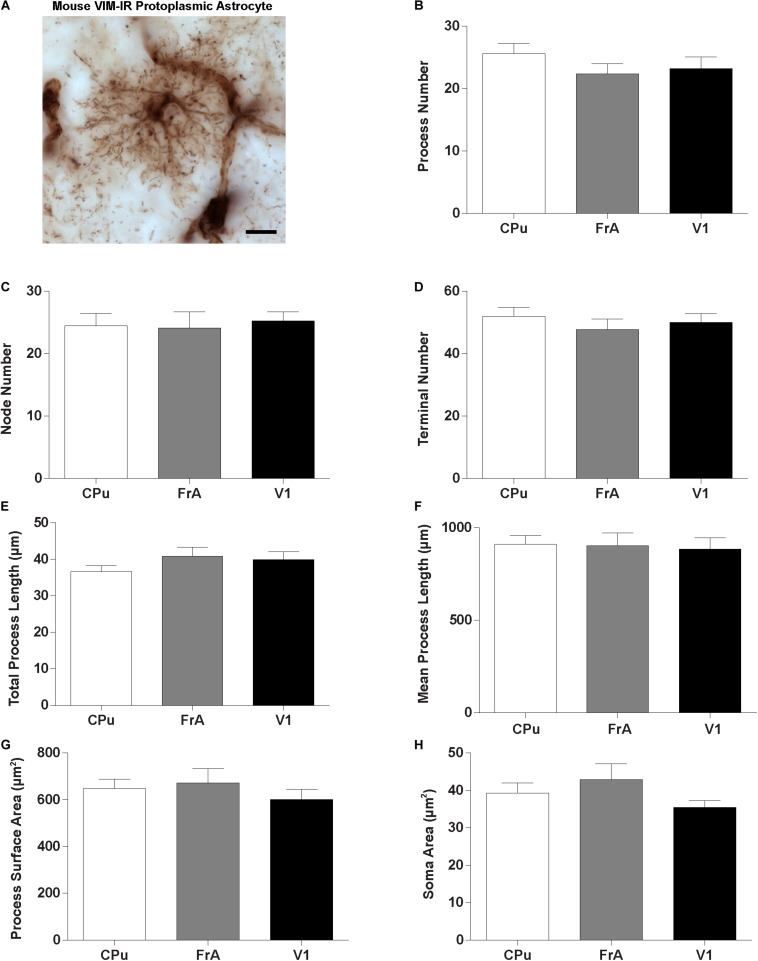
Lack of regional heterogeneity in mouse VIM-IR astrocyte morphometry. **(A)** Representative VIM-IR astrocyte from the mouse frontal association cortex gray matter. **(B–H)** In the mouse brain, comparing VIM-IR astrocytes between regions revealed no significant difference (*p* > 0.05) for any of the BSA. Scale bars = 10 μm (*n* = 5; Friedman Test).

Given the regional heterogeneity of human VIM-IR astrocytes, we then assessed whether morphological differences exist in three equivalent regions of the adult mouse brain. We did not assess the mouse mediodorsal thalamus due to its relatively small size and as it mostly lacked VIM-IR astrocytes similarly to what we observed in the human thalamus. Surprisingly, no regional differences were seen in the BSA measurements for VIM-IR astrocytes across these three mouse brain regions (Figure 7).

**FIGURE 8:**
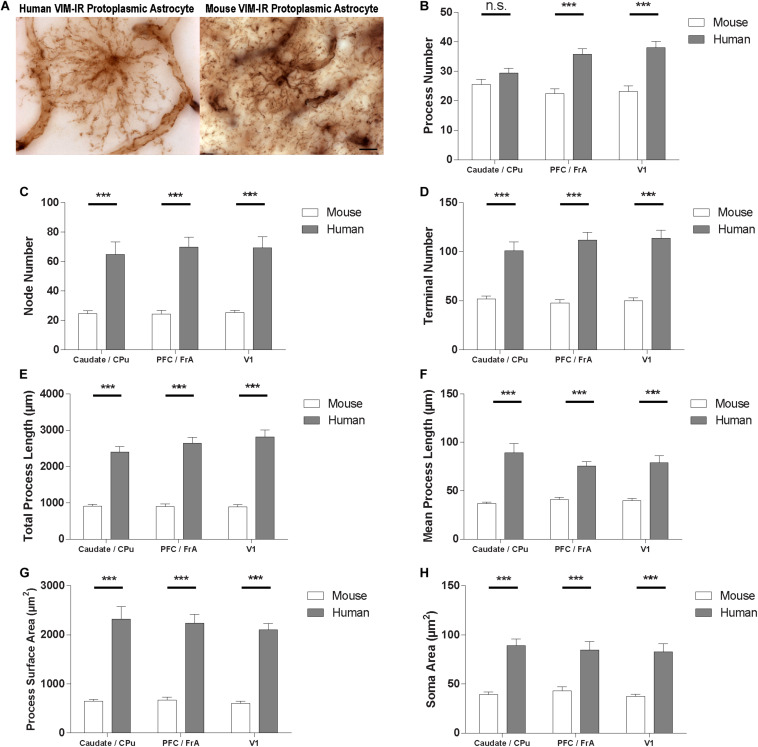
VIM-IR astrocyte morphometry reveals considerably more complex cerebral astrocytes in humans than in mouse. **(A)** Representative VIM-IR astrocytes from the human (left) and the mouse (right) primary visual cortex. **(B–H)** Human VIM-IR astrocytes were distinguishable from mouse VIM-IR astrocytes for all regions and BSA measures, except for process number in the caudate nucleus. Scale bars = 10 μm. n.s. *p* > 0.05, ***p ≤ 0.001 (*n* = 5; Mann–Whitney *U* test).

To quantify species differences in VIM-IR astrocyte morphometry, comparisons were made within these regions between BSA values for human and mouse VIM-IR astrocytes (Figure 8). This revealed human VIM-IR morphometry to be significantly more complex than mouse VIM-IR morphometry for all cases, except for process number in the striatum. The process number for human VIM-IR astrocytes was only 12% higher in the striatum (29 ± 2 vs. 26 ± 2 processes) but was 64% higher in the prefrontal cortex (36 ± 2 vs. 22 ± 2 processes) and 65% higher in the visual cortex (38 ± 2 vs. 23 ± 2 processes) (Figure 8B). The node number was 2.6-fold higher in the striatum (64.8 ± 8.6 vs. 24.5 ± 2.0 nodes), 2.9-fold higher in the prefrontal cortex (69.9 ± 6.7 vs. 24.1 ± 2.6 nodes), and 2.7-fold higher in the visual cortex (69.2 ± 7.5 vs. 25.2 ± 1.5 nodes) for human VIM-IR astrocytes relative to those in mouse (Figure 8C). The terminal number was almost 2-fold higher in the striatum (100.9 ± 9.0 vs. 51.9 ± 3.0 terminals), and 2.3-fold higher in both the prefrontal cortex (111.8 ± 7.8 vs. 47.8 ± 3.4 terminals) and visual cortex (113.8 ± 8.1 vs. 50 ± 2.8 terminals) for human VIM-IR astrocytes relative to those in mouse (Figure 8D). The total process length was 2.6-fold higher in the striatum (2396.4 ± 157.5 vs. 911.0 ± 47.8 μm), 2.9-fold higher in the prefrontal cortex (2642.1 ± 903.3 vs. 903.3 ± 69.4 μm), and 3.2-fold higher in the visual cortex (2819.7 ± 189.2 vs. 886.0 ± 61.1 μm) for human VIM-IR astrocytes relative to those in mouse (Figure 8E). The mean process length was 2.4-fold higher in the striatum (89.3 ± 9.6 vs. 36.6 ± 1.6 μm), 1.8-fold higher in the prefrontal cortex (75.5 ± 4.6 vs. 40.9 ± 2.5 μm), and 1.9-fold higher in the visual cortex (78.8 ± 7.6 vs. 39.9 ± 2.2 μm) for human VIM-IR astrocytes relative to those in mouse (Figure 8F). The process surface area was 3.5-fold higher in the striatum (2321.1 ± 251.9 vs. 648.0 ± 39.4 μm^2^), 3.3-fold higher in the prefrontal cortex (2237.7 ± 183.8 vs. 672.0 ± 60.1 μm^2^), and 3.5-fold higher in the visual cortex (2106.5 ± 128.4 vs. 601.3 ± 43.0 μm^2^) for human VIM-IR astrocytes relative to those in mouse (Figure 8G). The soma area was 2.3-fold higher in the striatum (89.1 ± 6.9 vs. 39.3 ± 2.6 μm^2^), 2-fold higher in the prefrontal cortex (84.5 ± 8.6 vs. 42.9 ± 4.2 μm^2^), and 2.4-fold higher in the visual cortex (82.9 ± 8.2 vs. 37.2 ± 2.4 μm^2^) for human VIM-IR astrocytes relative to those in mouse (Figure 8H). Together these morphometric comparisons reveal that human VIM-IR astrocytes are 2–3 fold greater in size than mouse VIM-IR astrocytes.

### Regional Vascular Density Correlates With GFAP-IR and VIM-IR Astrocyte Density

**FIGURE 9:**
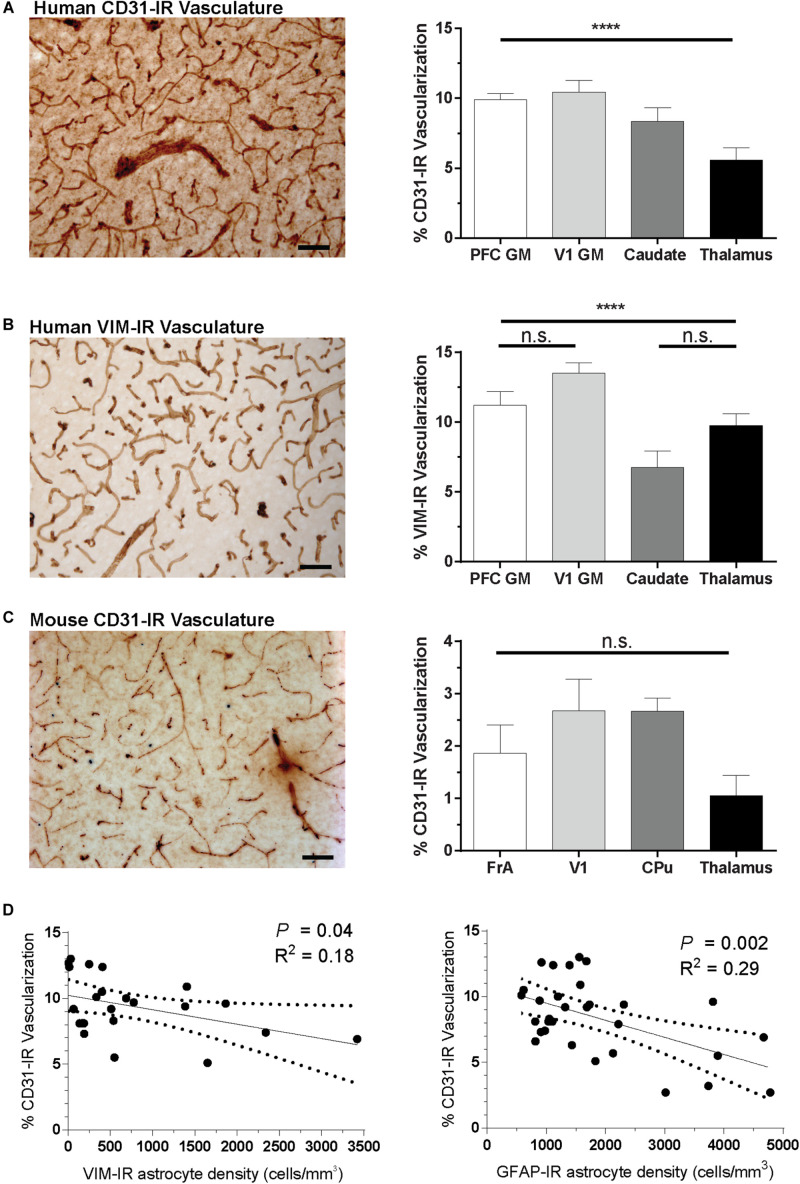
Regional differences in vascularization correlate with regional differences in astrocyte density. **(A,B)** The area occupied by CD31-IR and VIM-IR blood vessels was significantly greater in cortical than in subcortical regions. **(C)** There were no significant differences in the area occupied by CD31-IR blood vessels between mouse brain regions, but this coverage was substantially lower that that measured in human samples **(A,B)**. **(D)** Human VIM-IR and GFAP-IR astrocyte density negatively correlated with CD31-IR vascularization across regions. One value from the caudate nucleus was excluded as an outlier for both regressions, and both the thalamus and cortical white matter values were excluded from the VIM-IR density regression as VIM-IR cells were mostly absent from these regions. Scale bars = 50 μm. n.s. *p* > 0.05, ****p ≤ 0.0001 (Human: *n* = 8; Mouse: *n* = 5; Matched One-Way ANOVAs).

Given the abundance of VIM-IR astrocytes in close proximity to blood vessels in the caudate nucleus, we investigated a potential vascular basis for the regional heterogeneity of astrocytes by comparing the regional vascularization by CD31-immnoreactive (CD31-IR) or VIM-IR blood vessels in the same regions and subjects (Figure 9). In the same human and mouse subjects, significant regional differences were found for the area occupied by CD31-immunoreactive (CD31-IR) or VIM-IR blood vessels in human brain, but not for CD31-IR blood vessels in the mouse brain (Figure 9A–C). A significant negative correlation between regional CD31-IR vascularization and VIM-IR astrocyte density was found only when regions with a low VIM-IR astrocyte density (thalamus and cortical white matter) were excluded from the analysis (Figure 9D). A similar correlation was found between CD31-IR vascularization and GFAP-IR astrocyte density, however, this pattern occurred across all regions, indicating that highly vascularized human brain regions are generally less densely populated with VIM-IR or GFAP-IR astrocytes (Figure 9D).

## Discussion

To our knowledge, the present study is the first to anatomically assess the regional heterogeneity in density and morphology of astrocytes in the healthy human brain, to directly relate astrocytic and vascular densities, and to comprehensively compare human and mouse astrocytes. This study also provides the first quantitative description of VIM-IR astrocytes. Taken together, our results provide general patterns for astrocyte density, astrocyte morphology and vascular density across cortical and subcortical regions of the human brain.

### Vimentin Reliably Labels a Subset of Astrocytes in Adult Brain

As an astrocyte marker, VIM gives unprecedented access for visualizing the morphology of individual astrocytes, as it strongly reveals the fine morphology of a small, sparsely distributed population of astrocytes and its association with neighboring blood vessels. We also found VIM to be effective as a vascular marker, as it revealed a similar density of blood vessels as the conventional vascular marker CD31 in adult human brain, and both markers indicated that there is a significantly greater vascular density in cortical regions than in subcortical regions. VIM immunolabelling also revealed new subcortical structures. The presence of large, faintly labeled bundles of thin VIM-IR filaments of no clear cellular origin indicates either that VIM-IR astrocyte processes span long distances that often extend outside of the z-plane of a 50 μm-thick section, or that a vast network of VIM-IR fibers exist in the human brain. In addition, the common observation of VIM-IR twin cell morphology in the cortex and caudate nucleus suggests that VIM may have different roles than GFAP for cell division and differentiation in the adult human brain.

We found that most, but not all, VIM-IR cells co-express the commonly used astrocyte markers GFAP and Aldh1L1. We did not expect all VIM-IR cells to express GFAP, as not all mouse and human cortical astrocytes express GFAP (Sofroniew and Vinters, 2010; Sosunov et al., 2014), and the relatively frequent presence of twin cells in VIM IHC suggests that VIM may also label differentiating cells in the adult brain which are not mature GFAP-IR astrocytes. As in previous descriptions of GFAP-IR astrocytes, VIM labeling was localized to the soma and thick primary processes but not found in fine perisynaptic astrocytic processes (PAPs) lining the domains of protoplasmic astrocytes only visible with diolistic or transgenic labeling methods (Oberheim et al., 2009). Although Aldh1L1 is often reported to label all astrocytes, we found that only 85% of VIM-IR astrocytes also expressed Aldh1L1 in the mouse CPu. However, this particular transgenic Cre reporter line has previously been reported to show similar overlap (85–92% depending on the region chosen) with astrocytes immunolabelled with S100β, indicating that Aldh1L1 may not reveal the entire astrocyte population (Winchenbach et al., 2016). Nevertheless, the common coexpression of other astrocyte markers in VIM-IR cells indicates that VIM reliably labels astrocytes in adult human and mouse brains.

Most previous postmortem studies and animal studies have used GFAP as an astrocyte marker to implicate an abnormal distribution, density or morphology of astrocytes in psychiatric and neurological conditions (Brenner et al., 2001; Kim et al., 2018; Liddelow and Sofroniew, 2019). Of note, the regional expression of GFAP and the regional density of GFAP-IR astrocytes is consistently reduced in mood-associated brain regions in cases of major depressive disorder (MDD) and suicide (Torres-Platas et al., 2016; Nagy et al., 2017; Rajkowska et al., 2018). It would be interesting to examine whether the density of VIM-IR astrocytes is also reduced in samples from depressed patients. To date, however, more studies have associated VIM-IR astrocytes with neurological conditions. The first postmortem study of VIM-IR astrocytes qualitatively reported that they were hypertrophic in postmortem brain tissue from individuals with Pick’s Disease, multiple sclerosis (MS), amyotrophic lateral sclerosis (ALS), and Alzheimer’s Disease, and that VIM-IR astrocytes were almost exclusively associated with plaques labeled with β-amyloid protein (Yamada et al., 1992). Similarly, another postmortem study reported a reduced density of human VIM-IR astrocytes in the entorhinal cortex of individuals with Alzheimer’s Disease (Arnold et al., 1996). The only other postmortem immunohistochemical studies of human VIM-IR astrocytes have confirmed their presence in the brainstem, where, unlike GFAP-IR astrocytes, they have a reduced density in sudden unexpected death in epilepsy (Rusu et al., 2013; Patodia et al., 2019). Rodent studies have implicated that VIM protein can be released in astrocytic exosomes, and extracellular VIM protein promotes axonal regrowth in models of spinal cord injury (Shigyo and Tohda, 2016; Adolf et al., 2019). Together, these studies indicate the potential clinical importance of characterizing VIM-IR astrocytes in the healthy human brain.

### Regional Heterogeneity of VIM-IR Astrocyte Density and Morphology

Using an unbiased stereological approach, we found consistently fewer VIM-IR astrocytes than GFAP-IR astrocytes in all studied regions. These results alone indicate that VIM protein cannot be detectably expressed in all GFAP-IR astrocytes, as was also suggested using by FISH and coimmunofluorescence. We also show for the first time that VIM-IR and GFAP-IR astrocytes in subcortical regions can outnumber those in neocortical gray and white matter regions. This corresponds well with previous comparisons of regional GFAP mRNA levels and qualitative IHC observations (Torres-Platas et al., 2016), and may indicate that the human neocortex exhibits relatively limited regional heterogeneity of astrocyte density. Due to their particularly low number, pathological changes may be more readily observable for VIM-IR astrocytes than GFAP-IR astrocytes, and postmortem studies have already identified differences in the number and morphology of VIM-IR astrocytes in neurological conditions (Yamada et al., 1992; Arnold et al., 1996; Patodia et al., 2019). We found variety in subcortical astrocyte morphology, as VIM-IR caudate nucleus astrocytes had a protoplasmic-like morphology, whereas thalamic astrocytes had a fibrous-like morphology. A major finding, contrasting heavily with the very high density of GFAP-IR astrocytes in this region, was the almost complete absence of VIM-IR astrocytes in the human mediodorsal thalamus. This finding indicates that while many astrocytes co-express VIM and GFAP, GFAP can be expressed without VIM in at least one brain region. We also observed very few VIM-IR astrocytes in the mouse mediodorsal thalamus (not shown). This was unexpected given that the human mediodorsal thalamus has higher regional GFAP protein levels than many cortical regions (Torres-Platas et al., 2016). The lack of thalamic VIM-IR cells may indicate a regional difference in astrocyte division or differentiation, as early investigations indicated that immature astrocytes primarily express VIM until the time of cortical myelination, when they mature by switching to primarily expressing GFAP (Dahl, 1981; Pixley and de Vellis, 1984). The absence of thalamic VIM-IR cells also indicates that thalamic astrocytes have a particular cytoskeletal organization, as mouse astrocytes require VIM, but not GFAP, to synthesize other astrocyte-enriched type III intermediate filaments (Pekny et al., 1999; Jing et al., 2007). Finally, the absence of VIM might affect the functional efficiency of mediodorsal thalamic astrocytes, as intermediate filaments increase the rate of chemokine-induced vesicular trafficking of astrocytes *in vitro* (Vardjan et al., 2012). These findings may not generalize to other thalamic nuclei, as there are large differences in GFAP-IR astrocyte density across thalamic nuclei in mouse (Emsley and Macklis, 2006). Nevertheless, a recent study has shown that astrocyte activity coordinated with local neurons in the mediodorsal thalamus is involved in initiating cortical blood-oxygen-level-dependent responses (Wang et al., 2018). On this basis we tentatively speculate that, as for neurons, astrocytes may have different functional roles in the thalamus than in the striatum or neocortex.

All measured morphometric features of VIM-IR astrocytes varied significantly across regions, which further supports region-specific functions for VIM-IR astrocytes. For instance, we qualitatively and quantitatively observed astrocytes in the caudate nucleus to have a protoplasmic-like morphology, whereas those in the mediodorsal thalamus to have a fibrous-like morphology. To our knowledge this is one of the first quantitative indications of morphological diversity for non-cortical astrocytes in the human brain. Additionally, while human protoplasmic and fibrous cortical astrocytes are usually defined qualitatively by their location and morphology, previous studies have not clearly provided a quantitative basis for the fundamental morphological distinction (Oberheim et al., 2009; Torres-Platas et al., 2011). Here, our results indicate that VIM-IR protoplasmic and fibrous astrocytes in the human cortex are most reliably distinguished by terminal number and branching than by the number, length or surface area of processes. This quantitative distinction should inform future studies on cortical human astrocytes, especially in experiments where astrocytes are not clearly situated in gray or white matter.

To complement our morphological findings, we compared the complexity and diversity of VIM-IR protoplasmic astrocytes in equivalent gray matter regions of the human and mouse brain. As expected, based on previous reports for GFAP-IR astrocytes (Oberheim et al., 2006, 2009), VIM-IR astrocyte morphology was considerably more complex in human than in mouse for all features and regions studied, except for process number in the caudate nucleus. The latter does not signify an important region-specific species difference, as the morphometric species differences for striatal VIM-IR astrocytes were similar to those for cortical VIM-IR astrocytes for all other features. Interestingly, no regional differences were found for mouse VIM-IR astrocyte morphometry, suggesting that VIM-IR astrocytes are more regionally diverse in the human brain.

### VIM-IR and GFAP-IR Astrocyte Density Inversely Correlates With Vascular Density

We extended our species comparisons to regional vascularization and found that human brain regions were 2– to 5–fold more vascularized than equivalent mouse brain regions. Both VIM and CD31 showed significant differences between human subcortical regions and cortical regions, by contrast, CD31 showed no significant differences between mouse brain regions. While the vascular density of these human brain regions has not, to our knowledge, been reported before, our measurements for mouse brain vascular density are similar to previous estimates using more precise methods (Chugh et al., 2009).

Given our observation of regional heterogeneity of VIM-IR astrocyte morphometry and vascularization in the human brain, but not in mouse brain, we then assessed whether these were quantitatively related. While no correlation was found for any human VIM-IR astrocyte morphometric features and vascularization (not shown), we found that VIM-IR astrocyte density inversely correlated with regional vascular density in cortical gray matter and the caudate nucleus (but not in the thalamus or cortical white matter). By comparison, GFAP-IR astrocyte density inversely correlated with vascular density for all studied regions, suggesting that gliovascular interactions play a more consistent role distributing GFAP-IR astrocytes than VIM-IR astrocytes throughout the human brain. Gliovascular interactions may be relatively robust for GFAP-IR astrocytes, given that depressed individuals have altered vascular coverage for astrocytes labeled by aquaporin-4, but not for those labeled by GFAP (Rajkowska et al., 2013). A more precise analysis of vascular contacts of VIM-IR and GFAP-IR astrocytes across regions may reveal whether this inverse correlation between astrocyte and vascular density is mediated by regional alterations in gliovascular interactions, perhaps in the number or volume of vascular endfeet contacts per astrocyte.

### Limitations and Future Directions

To our knowledge this is the most systematic morphological and stereological investigation of astrocytes in the human brain to date. However, this study presents two main limitations: its relatively limited sample size, which underpowered some statistical comparisons, and the fact that only samples from males were analyzed. Although a total of 180 VIM-IR astrocytes were manually traced in this study, this meant only four representative cells per region per subject, which constitutes a limitation in the statistical power of morphometric comparisons made in this study. Despite this, we are confident that the strong effect sizes observed reflect important regional and cross-species trends in astrocyte morphometry. While sex differences have been reported for astrocyte density and morphology in the mouse amygdala (Johnson et al., 2008), and for transcriptional changes in prefrontal cortex astrocytes in depressed patients and stressed mice (Labonté et al., 2017), sex differences have yet to be reported for astrocytes in the healthy adult brain. Including different age groups would also have helped elucidate the effect age has on VIM-IR and GFAP-IR astrocytes in healthy human brain, given that cerebral GFAP is known to increase with age in mice and humans (Boisvert et al., 2018; Wruck and Adjaye, 2020). We were unable to assess these differences mostly because of the limited availability of matched female samples, but also because of the small sample size suitable for an extensive cross-regional comparison which would have been difficult to achieve robust comparisons. Future studies should examine potential sex differences and further explore the functional consequences associated with regional differences in astrocytic densities and morphologies. Such knowledge, together with the data presented in this study, will be useful for studies examining astrocytes in cerebral pathologies.

## Data Availability Statement

The datasets generated for this study are available on request to the corresponding author.

## Ethics Statement

The studies involving human participants were reviewed and approved by Douglas Hospital Research Ethics Board of the Douglas Mental Health University Institute. The patients/participants provided their written informed consent to participate in this study. The animal study was reviewed and approved by McGill University’s Animal Care Committee (MACC approval ID: 5473), and Montreal General Hospital Animal Use Protocol (ID: 6005).

## Author Contributions

LO’L and NM conceived the project, designed the experiments, and prepared the manuscript. LO’L, CB, AT, and MD performed the immunolabelling experiments. LO’L and JM carried out the stereological cell counts. LO’L performed the morphometric and statistical analyses. WF and KM contributed to the mouse experiments, including providing transgenic mouse brains. All the authors contributed to the interpretation of the results in addition to participating in the finalization of the manuscript.

## Conflict of Interest

The authors declare that the research was conducted in the absence of any commercial or financial relationships that could be construed as a potential conflict of interest.
